# Comparative study of urea-^15^N fate in pure bamboo and bamboo-broadleaf mixed forests

**DOI:** 10.3389/fpls.2024.1382934

**Published:** 2024-05-21

**Authors:** Yiyuan Wu, Wenyuan Dong, Huan Zhong, Jixia Duan, Weidong Li, Chan Pu, Xin Li, Zexuan Xie

**Affiliations:** ^1^ College of Forestry, Nanjing Forestry University, Nanjing, China; ^2^ Institute of Qiong Bamboo, Southwest Forestry University, Kunming, China; ^3^ Key Laboratory of State Forestry Administration on Biodiversity Conservation in Southwest China, Southwest Forestry University, Kunming, China; ^4^ College of forestry, Southwest Forestry University, Kunming, China; ^5^ Daguan County Forestry and Grassland Bureau, Zhaotong, China

**Keywords:** biomass, N recovery efficiency, bamboo-broadleaf mixed forests, *Qiongzhuea tumidinoda*, ^15^N tracing technology

## Abstract

**Objectives:**

Bamboo is a globally significant plant with ecological, environmental, and economic bene-fits. Choosing suitable native tree species for mixed planting in bamboo forests is an effective measure for achieving both ecological and economic benefits of bamboo forests. However, little is currently known about the impact of bamboo forests on nitrogen cycling and utilization efficiency after mixing with other tree species. Therefore, our study aims to compare the nitrogen cycling in pure bamboo forests with that in mixed forests.

**Methods:**

Through field experiments, we investigated pure *Qiongzhuea tumidinoda* forests and *Q. tumidinoda-Phellodendron chinense* mixed forests, and utilized ^15^N tracing technology to explore the fertilization effects and fate of urea-^15^N in different forest stands.

**Results:**

The results demonstrated the following: 1) in both forest stands, bamboo culms account for the highest biomass percentage (42.99%-51.86%), while the leaves exhibited the highest nitrogen concentration and total nitrogen uptake (39.25%-44.52%/29.51%-33.21%, respectively) Additionally, the average nitrogen uptake rate of one-year-old bamboo is higher (0.25 mg kg^-1^ a^-1^) compared to other age groups. 2) the urea-^15^N absorption in mixed forests (1066.51–1141.61 g ha^-1^, including 949.65–1000.07 g ha^-1^ for bamboo and 116.86–141.54 g ha^-1^ for trees) was significantly higher than that in pure forests (663.93–727.62 g ha^-1^, *P<0.05*). Additionally, the ^15^N recovery efficiency of culms, branches, leaves, stumps, and stump roots in mixed forests was significantly higher than that in pure forests, with increases of 43.14%, 69.09%, 36.84%, 51.63%, 69.18%, 34.60%, and 26.89%, respectively. 3) the recovery efficiency of urea-^15^N in mixed forests (45.81%, comprising 40.43% for bamboo and 5.38% for trees) and the residual urea-^15^N recovery rate in the 0–60 cm soil layer (23.46%) are significantly higher compared to those in pure forests (28.61%/18.89%). This could be attributed to the nitrogen losses in mixed forests (30.73%, including losses from ammonia volatilization, runoff, leaching, and nitrification-denitrification) being significantly lower than those in pure forests (52.50%).

**Conclusion:**

These findings suggest that compared to pure bamboo forests, bamboo in mixed forests exhibits higher nitrogen recovery efficiency, particularly with one-year-old bamboo playing a crucial role.

## 1 Introduction

*Qiongzhuea tumidinoda* was one of the two bamboo species listed in the first edition of the “Chinese Rare and Endangered Plants Protection List” published in 1984, designated as a nationally protected plant at the third level. It is indigenous to the southwestern region of China, with its natural distribution confined to a narrow strip along the lower reaches of the Jinsha River in the provinces of Sichuan and Yunnan. There exists a total area of 13900 hectares of natural *Q. tumidinoda* resources in Daguan County. This area accounts for 59% of the global total area of natural *Q. tumidinoda*, which amounts to 23560 hectares (Dong, 2019; Wu et al., 2023b). The highly raised nodes on the culms of *Q. tumidinoda* make it an excellent material for crafting walking sticks, bamboo handicrafts, and round bamboo furniture (Yiyuan et al., 2020; Li et al., 2021). Bamboo shoots from *Q. tumidinoda* are renowned for their exquisite taste, crisp and tender texture, and delightful sweetness, while also boasting a rich nutritional profile. Consequently, over 90% of products derived from *Q. tumidinoda*, such as fresh bamboo shoots, dried bamboo shoots and salted bamboo shoots, have consistently enjoyed robust sales in Japan and the Greater China region, encompassing Hong Kong, Macau, and Taiwan (Dong, 2019; Li et al., 2021).

*Q. tumidinoda* demonstrates rapid growth, taking just around 50 days from the emergence of bamboo shoots to reaching full height and diameter, enabling the proliferation of numerous new individuals within a span of two months and consuming substantial nutrients (Dong et al., 2002; Wu et al., 2022). *Q. tumidinoda* forests are primarily managed for bamboo timber and bamboo shoots. With the rapid development of the bamboo industry, the substantial annual harvest of bamboo timber and bamboo shoot biomass inevitably leads to direct removal of a significant amount of nutrients, resulting in the depletion of soil nutrients in bamboo forests (Yang et al., 2012; Li et al., 2024). Furthermore, the slow decomposition of residual rhizomes and stumps left after harvesting in bamboo forests results in a low nutrient return rate (Jiang, 2007; Zheng et al., 2022). Therefore, achieving sustainable high yields in bamboo forests requires nutrient supplementation through fertilization. Among these, nitrogen fertilizer stands as the primary nutrient factor enhancing bamboo forest productivity (Sardar et al., 2023; Zhao and Cai, 2023; Zou et al., 2023). In bamboo forest ecosystems, nitrogen allocation directly influences the growth of bamboo shoots, the development of bamboo culms, and the overall productivity of the stand (Wu et al., 2023a; Zuo et al., 2024).

However, overreliance solely on nitrogen fertilizers can lead to a series of issues such as soil compaction and groundwater contamination, particularly pronounced in monoculture bamboo forests (Tariq et al., 2015; Wang et al., 2023a). To tackle this challenge, strategies involving intercropping broadleaf forests with bamboo stands are frequently employed (Peng et al., 2021). Research indicated that compared to pure forests, mixed forests may have had higher species diversity, leading to potentially more diverse root exudates and leaf litter, further enhancing soil chemical properties such as total nitrogen (Gillespie et al., 2021; Liang et al., 2022). Additionally, studies found differences in microbial diversity and composition between pure and mixed forests, resulting in distinct nitrogen utilization patterns possibly indirectly influenced by pH and differing litter qualities (Wen et al., 2014; Bai et al., 2023). These variances possibly led to higher rates of soil nitrogen mineralization and nitrification in broadleaf trees in mixed forests compared to pure ones, consequently elevating nitrogen concentrations in the soil (Yan et al., 2008; Kong et al., 2020; Yan et al., 2022). Furthermore, broadleaf forests aid in soil moisture retention, providing compensatory ecosystem services to address the limitations of pure bamboo forest ecosystems (Bauhus et al., 2017; Gong et al., 2022). Research has shown that this difference was determined by specific hybrid tree species and hybrid ratios. For instance, Weih et al (Weih et al., 2021). studied the nitrogen utilization patterns of four hybrid willow forests and found that individual species’ functionalities played a determining role. Recent studies on nitrogen in monoculture and mixed forests focused mostly on the distribution patterns of nitrogen in plants, soil or systems, but there was limited research on the fate of nitrogen in monoculture and mixed forests (Voigtlaender et al., 2019; Masuda et al., 2022).

*Q. tumidinoda*, a small to medium-sized bamboo species, thrives in temperate and humid environments. Lots of research have indicated that mixed forests of *Q. tumidinoda* with broadleaf trees exhibit superior productivity (Zhang et al., 2020b; Chen et al., 2022), soil quality (Xia et al., 2022; Yuan et al., 2022), species diversity (Yang et al., 2012) and water conservation (Zhong et al., 2020) compared to pure *Q. tumidinoda* forest. Studies on nitrogen in *Q. tumidinoda* have mainly focused on soil nitrogen concentration related to its growth (Zhang et al., 2020b; Zhong et al., 2023), whereas research on nitrogen allocation patterns in bamboo and its utilization in different forest types remains unexplored. Simultaneously, to effectively utilize nitrogen, the ^15^N tracing technique has been widely employed to quantify nitrogen fertilizer uptake, residual amounts, and losses in research. However, few studies have existed regarding the distribution and translocation of urea-^15^N in different forest types within *Q. tumidinoda* ecosystems. This study focuses on bamboo and soil from *Q. tumidinoda* forests and mixed forests of *Q. tumidinoda* with *P. chinense*. Utilizing ^15^N tracing techniques, the objectives are: (1) to compare the partitioning efficiency of applied nitrogen in different organs at various ages between pure forests and mixed forests; (2) to compare the nitrogen recovery and residue rates in bamboo ecosystems between pure forests and mixed forests to determine which type exhibits higher rates.

## 2 Materials and methods

### 2.1 Site description

**Figure 1:**
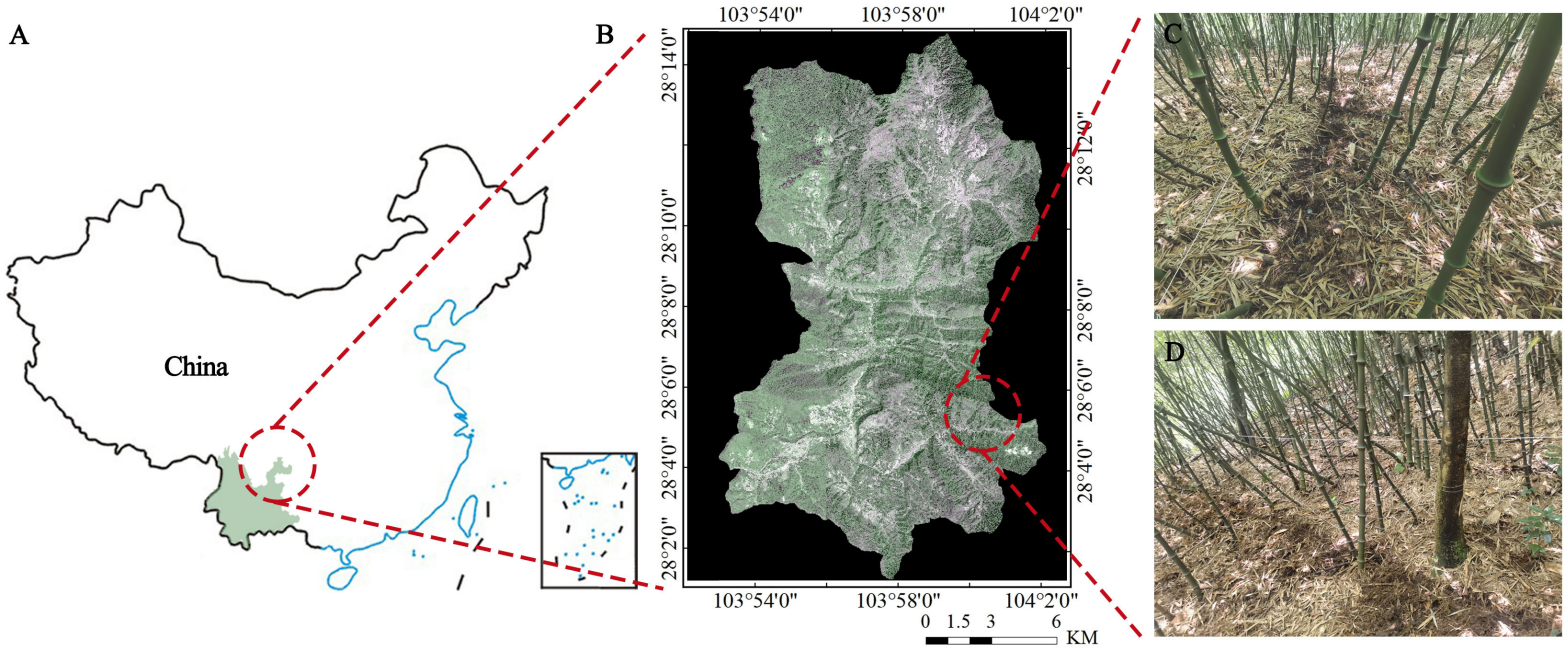
Geographical location of the research area. **(A)** China; **(B)** Daguan County, Zhaotong city; **(C, D)** Q and Q-P represent a mixed forest of pure *Q. tumidinoda* and *Q. tumidinoda-Phellodendron chinense*, respectively.

The experimental site is located in Daluohanba, Mugan Town, Daguan County, Yunnan Province, China (**Figure 1**), the climate condition is the moderate temperate continental climate, with an annual average temperature of 10.5°C, the maximum temperature of 29°C, the lowest temperature of -10°C, annual average precipitation of 1200 mm, annual average evaporation of 1076 mm and relative humidity of 85%. The soils in the research area were a type of yellow-brown forest soil (mostly Inceptisols, United States Soil Taxonomy), originating from basalt with a loam texture. Before the experiment, we measured the soil physical properties of the 0–60 cm soil depth. The soil physicochemical properties were presented in **Table 1**, soil bulk density was measured by the ring knife method (Qiao et al., 2020). Soil organic matter was determined by the potassium dichromate external heating method (Fan et al., 2016). Soil pH was determined using a pH meter at a soil/water ratio of 1:2.5. Soil total nitrogen (TN) was determined by the appropriate Kjeldahl’s method. soil total phosphorus (TP) and total potassium (TK) were determined by using colorimetrically (ammonium molybdate method) and flame photometer after wet digestion (Bao, 2000).

**Table 1:** Soil physicochemical properties in soil layer 0–60 cm across various forest types (Mean ± SD).

Forest type	Soil depth (cm)	Bulk density (g cm^-3^)	pH (1:2.5)	Organic matter (g kg^-1^)	Total N (g kg^-1^)	Total P (g kg^-1^)	Total K (g kg^-1^)
Q	0–20	1.12 ± 0.04	4.76 ± 0.32	77.55 ± 5.63	5.27 ± 0.61	0.61 ± 0.04	25.04 ± 1.23
	20–40	1.31 ± 0.08	5.36 ± 0.35	43.10 ± 3.40	2.09 ± 0.46	0.46 ± 0.02	21.09 ± 1.81
	40–60	1.41 ± 0.07	5.79 ± 0.41	25.74 ± 1.65	1.58 ± 0.39	0.39 ± 0.03	18.63 ± 0.99
Q-P	0–20	1.02 ± 0.09	4.85 ± 0.28	91.66 ± 6.19	6.20 ± 33.79	0.93 ± 0.07	33.38 ± 2.65
	20–40	1.18 ± 0.06	5.37 ± 0.26	51.44 ± 2.41	2.74 ± 0.25	0.77 ± 0.05	26.10 ± 1.85
	40–60	1.30 ± 0.06	5.89 ± 0.34	32.18 ± 2.62	1.98 ± 0.18	0.67 ± 0.04	20.24 ± 1.74

There were two types of forest stands in the study area, Q: *Q. tumidinoda* pure forest and Q-P: a mixed forest of *Q. tumidinoda* and artificially planted 1-year-old saplings of *Phellodendron chinense.* The *Q. tumidinoda* forest was a natural forest, The *P. chinense* was planted in September 2012 within the bamboo forest at a density of 400 individuals per hectare, with a spacing of 5m × 5m between plants, an average diameter at breast height (DBH) of 6.12 cm, and an average tree height of 5.50 m. Underneath the forest canopy, there are understory plants including *Hydrangea davidii*, *Smilax china*, *Elatostema involucratum*, *Selaginella tamariscina*, *Achyranthes bidentata*, *Pilea sinofasciata*, and *Dryopteris erythrosora* and the vegetation cover is approximately 30%.

### 2.2 Experimental design

The experimental design employed a factorial design with two forest types (Q:28° 5′ N, 104° 0′ E, altitude 1488 - 1512 m a. s. l., slope 21, Q-P:28° 6′ N, 104° 1′ E, altitude 1411 - 1435 m, a. s. l., slope 22°), and two treatments: fertilized and unfertilized. The experimental plots had an area of 400 m^2^ (20 m × 20 m), replicated three times, with distances larger than 20 m between adjacent plots. Four isolation trenches were excavated around each plot, with a depth of 60 centimeters to sever rhizomes and effectively prevent long-distance nutrient transport. The bamboo in the study area was used for both shoot harvesting and timber production. Before the initiation of the experiment, uniform density control measures were applied to the bamboo forest; however, fertilization management was not implemented. The bamboo stand structures before harvest for the two forest types are presented in **Table 2**.

**Table 2:** Bamboo forest structure (Mean ± SD).

Forest type	Bamboo forest area (ha^-1^)	Density (individual ha^−1^)	Mean DBH (cm)	Mean Height (m)	Age Structure (1a:2a:3a:4a)
Q	3.12	77800 ± 1415	1.41 ± 0.40	4.18 ± 0.74	1.28:1.56:1.72:1
Q-P	3.05	76000 ± 566	1.58 ± 0.42	4.59 ± 0.83	1.44:1.56:1.66:1

1a, 2a, 3a and 4a represent 1, 2, 3 and 4 years, respectively.

Per the previous study (Wang et al., 2022), the experimental fields received a single application of 300 kg ha^−1^ urea (46% N). Additionally, in each fertilization plot, 200 g of ^15^N-labeled urea (10.06 atom%, supplied by the Shanghai Research Institute of Chemical Industry) was administered. The fertilization experiment was conducted in August 2022 (Dai et al., 2011), coinciding with the initiation of substantial underground growth in the bamboo, demanding a significant nutrient supply. Furrow application was employed in the trial, consisting of nine fertilizer furrows per plot arranged along contour lines (each furrow measuring 0.2 meters in width, 0.15 meters in depth, spaced 2 meters apart). Before application, all fertilizers were thoroughly mixed and uniformly blended, then applied at the specified depth. To achieve this, initially blend 200 g of ^15^N-labeled urea with 2 kg of urea evenly, subsequently distribute 10 kg of urea uniformly to a specific depth, sprinkle 2.20 kg of the mixed urea evenly on its surface, turn over and uniformly mix.

### 2.3 Plant and soil sampling and analyses

In each plot, three bamboo individuals with different ages (1a, 2a, 3a, 4a) and with an average diameter at DBH were selected and harvested, totaling 144 individuals in November 2022. The bamboo was separated into culms, branches, leaves, stumps, and stump roots. The specific procedure was as follows: Firstly, the diameter of each bamboo was measured one by one using a vernier caliper. Next, standard bamboo specimens were selected and cut down, and their heights were measured using a steel tape measure. Subsequently, all leaves and branches were collected, the bamboo culms were segmented and labeled, the stumps were excavated, and allstump roots were collected, cleaned, dried, and labeled. The sampling method for rhizomes and rhizome roots involved placing five randomly selected 1 m × 1 m subplots in an “S” shape within each plot. All culms and culm roots were collected, washed, dried, and labeled, and then their fresh weights were measured in batches. Finally, each organ (rhizomes sampled in appropriate proportions) was taken back to the laboratory, where fresh samples were dried at 105°C, then dried at 70°C to constant weight to determine dry weight and calculate organ biomass. m m Dried samples were ground and sieved through a 0.15 mm mesh screen for ^15^N analysis.

An Isotope Ratio Mass Spectrometer (IsoPrime 100, IsoPrime limited, UK) was employed to analyze the total nitrogen content in all plant and soil samples, the pure abundance of nitrogen in both plant and soil from the unfertilized plot, and the atom percentage of ^15^N in the fertilized plot.

### 2.4 Calculation methods

The urea-^15^N derived percentage (%Ndff) is calculated using Equation 1, while other nitrogen-related indicators are calculated separately using Equations 2–7. (Shi et al., 2012; Su et al., 2019):


(1) $$\text{\%Ndff}=\frac{b-a}{c-a}\times 100$$

In which a is the at% ^15^N in the unfertilized plant organ or soil, b represents the atom% ^15^N of the fertilized plant organ or soil, and c is the atom% ^15^N of the fertilizer.


(2) $$\text{Organ total uptake N}\left(\text{kg h}{\text{a}}^{-1}\right)=\text{organ dry matter }\left(\text{t h}{\text{a}}^{-1}\right)\times \text{N concentration}\left(\text{g k}{\text{g}}^{-1}\right)\times 10^{-6}$$

(3) $$\text{Organ}{\;}^{15}\text{N uptake}\left(\text{kg h}{\text{a}}^{-1}\right)=\left(2\right)\times \left(1\right)\times 10^{-2}$$

(4) $$\text{Plant}{\;}^{15}\text{N uptake}\left(\text{kg h}{\text{a}}^{-1}\right)=\sum \left(3\right)$$

(5) $$\begin{array}{l} \text{Urea}{-}^{15}\text{N residual of soil }\left(\text{kg h}{\text{a}}^{-1}\right)=\left(\text{fertilization area}\left({\text{m}}^{2}\right)\times \text{soil thickness}\left(\text{cm}\right)\right.\\ \times \text{ soil bulk density }\left(\text{g c}{\text{m}}^{-3}\right)\times \text{N concentration}\left(\text{g k}{\text{g}}^{-1}\right)\times \left(1\right)\times 10^{-2})/400\left({\text{m}}^{2}\right) \end{array}$$

(6) $$\text{N recovery efficiency }\left(\%\right)=\left(4\right)/\text{Total fertilizer}{\;}^{15}\text{N}\left(\text{kg h}{\text{a}}^{-1}\right)\times 100$$

(7) $$\begin{array}{l} \text{N residual efficiency }\left(\%\right)=\left(5\right)/\text{Total fertilizer}{\;}^{15}\text{N}\left(\text{kg h}{\text{a}}^{-1}\right)\times 100\\ \text{ N loss efficiency }\left(\%\right)=1–\left(6\right)–\left(7\right) \end{array}$$

### 2.5 Statistical analysis

One-way analysis of variance (ANOVA) was employed to assess significant distinctions among treatments across all variables throughout the experiment. The Duncan’s multiple range test was utilized for mean separation, and statistical significance was determined at *P< 0.05*. by SPSS 23.0 (SPSS Inc., Chicago, IL, USA), while figure creation relied on Origin 8.6 software (OriginLab Corporation, Northampton, MA, USA).

## 3 Results

### 3.1 *Q. tumidinoda* bamboo biomass

**Figure 2:**
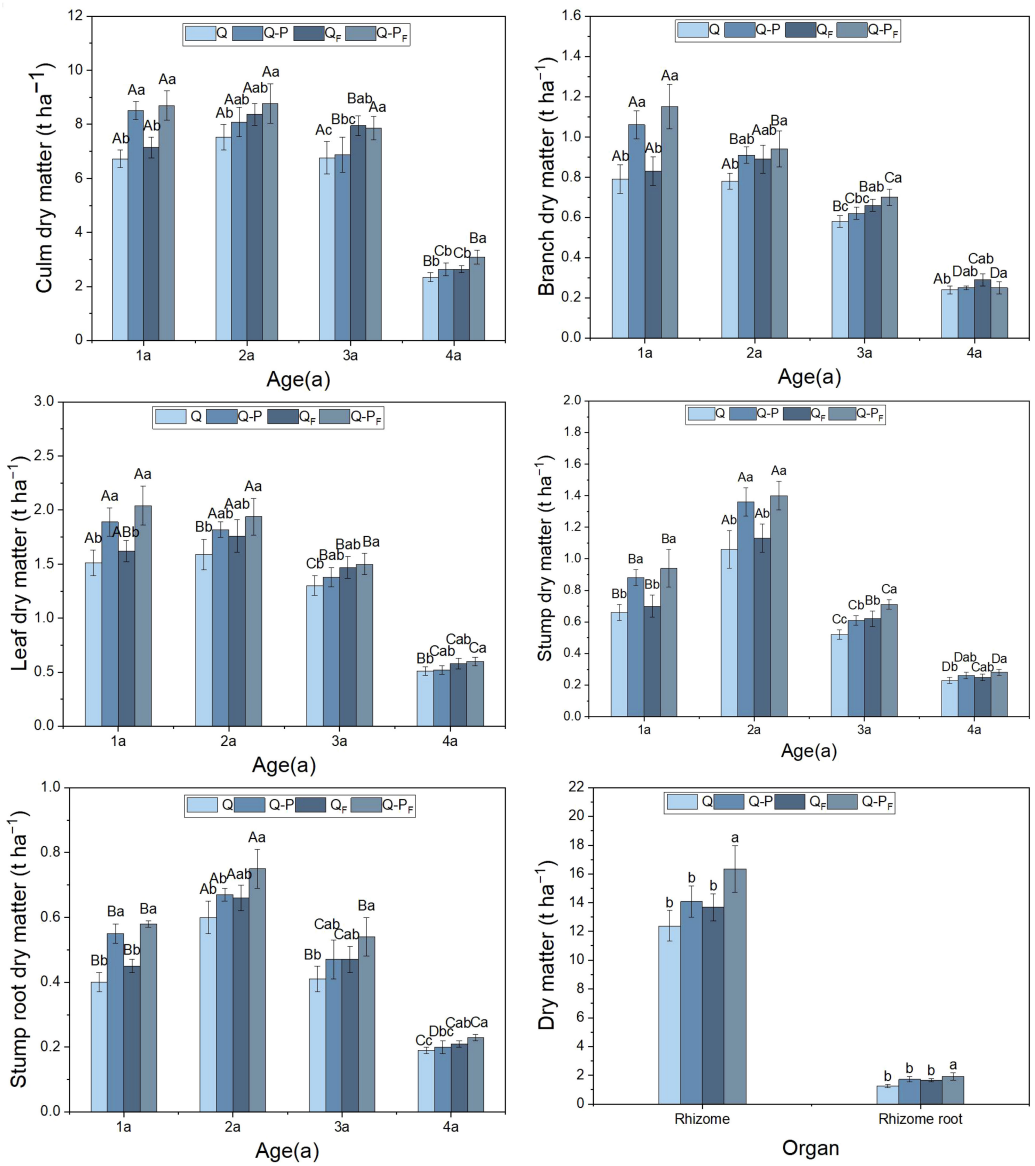
Organ biomass characteristics of *Qiongzhuea tumidinoda* forests across four types of plots (Mean ± SD). In the first five figures, different uppercase letters in the same sampling site represent significant distinctions between different ages (*P< 0.05*), and different lowercase letters in the same age indicate significant differences between sampling sites (*P< 0.05*). In the last figure, different lowercase letters within the same organ indicate significant differences between different plots (*P< 0.05*).

Under fertilization treatment, the biomass of various organs in bamboo of Q-P type at different ages was significantly higher than that of Q type (**Figure 2**, *P< 0.05*). This result was similar under no fertilization treatment, but the difference in rhizomes and rhizome roots were not significant (P*>0.05*). Compared with the unfertilized treatment, under the fertilization treatment, the biomass of various organs in bamboo of different ages of Q-P and Q types increased, but only the difference in rhizomes and rhizome root of Q-P type bamboo reached a significant level. The average proportion of bamboo culm biomass to total biomass was 47.55% (ranging from 42.99% to 49.33%), ranking first. Following that, the rhizomes emerged as the second-highest component, with an average biomass of 14.12 t/ha^-1^ and peaking at 17.93 t/ha^-1^ in Q-P_F_ type, with no significant differences observed in other plots (*P > 0.05*). The aboveground biomass of the bamboo in the four plots (comprising leaves, branches, and culms) exceeded the belowground biomass (including stumps, stump roots, rhizomes, and rhizome roots).

### 3.2 N Concentration and N uptake

**Figure 3:**
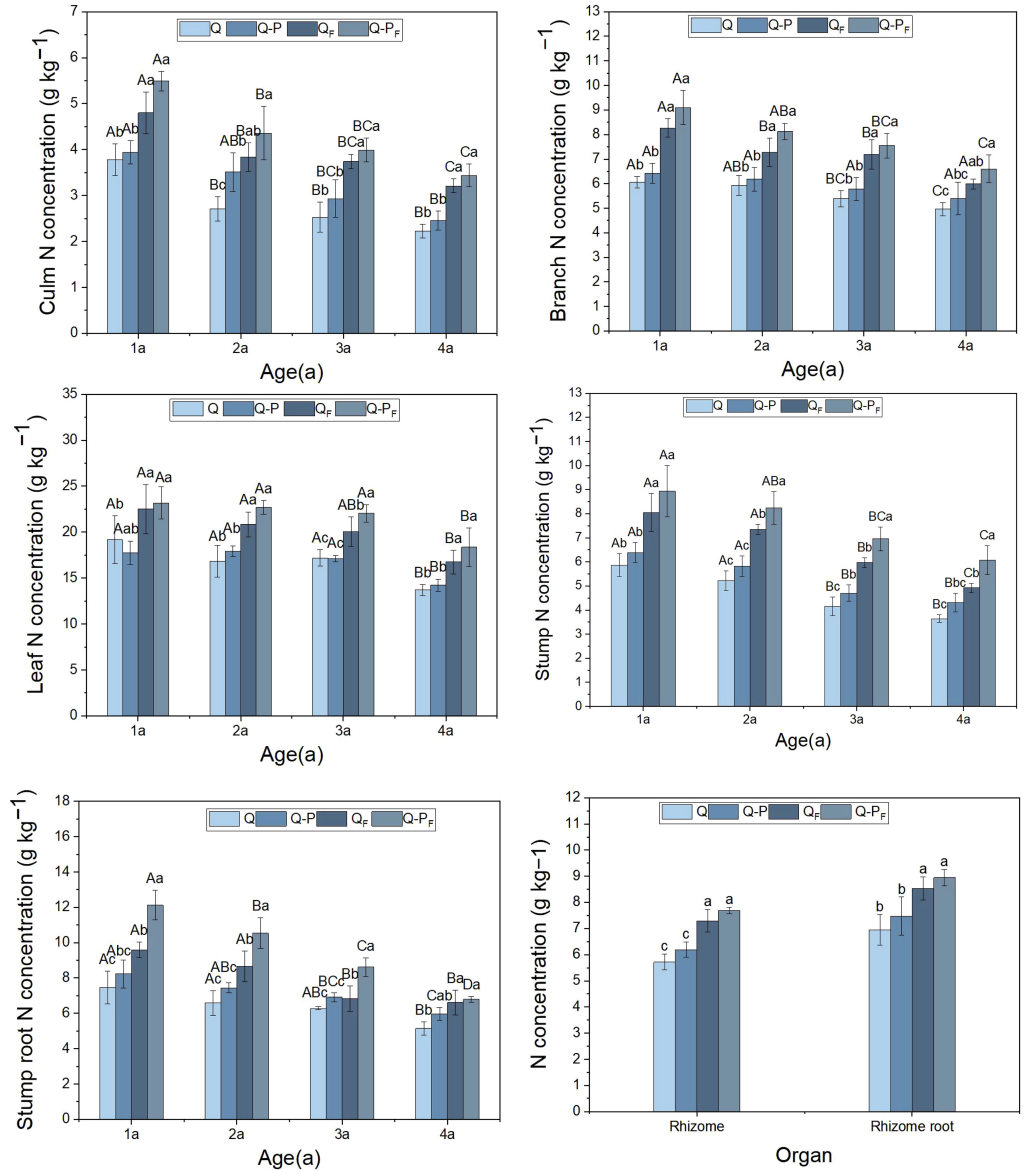
Organ nitrogen concentration of *Qiongzhuea tumidinoda* forests across four types of plots (Mean ± SD). In the first five figures, different uppercase letters in the same sampling site represent significant distinctions between different ages (*P< 0.05*), and different lowercase letters in the same age indicate significant differences between sampling sites (*P< 0.05*). In the last figure, different lowercase letters within the same organ indicate significant differences between different sampling plots (*P< 0.05*).

Under the fertilization treatment, the nitrogen concentrations in various organs of different-aged bamboo in the *Q. tumidinoda* mixed forest (Q-P) were higher than those in the pure *Q. tumidinoda* forest (Q, **Figure 3**). This result was similar to the unfertilized treatment, but some organs showed no significant differences (*P > 0.05*). Compared to the unfertilized treatment, the nitrogen concentrations in various organs of Q-P and Q types significantly increased under the fertilization treatment, with Q-P type (averaging a growth of 41.92%) showing a more pronounced increase than Q type (averaging a growth of 37.23%). Among the types, Q-P_F_ type exhibited the highest concentrations in various organs, followed by Q_F_ type. The nitrogen concentrations varied among different organs of *Q. tumidinoda*, with the leaves of bamboo in all four plots exhibiting significantly higher nitrogen concentrations than other organs, ranging from 13.76 to 27.03 g kg^-1^. Additionally, the nitrogen concentrations in various organs across the four plots showed a decreasing trend with the increasing age of the bamboo.

**Figure 4:**
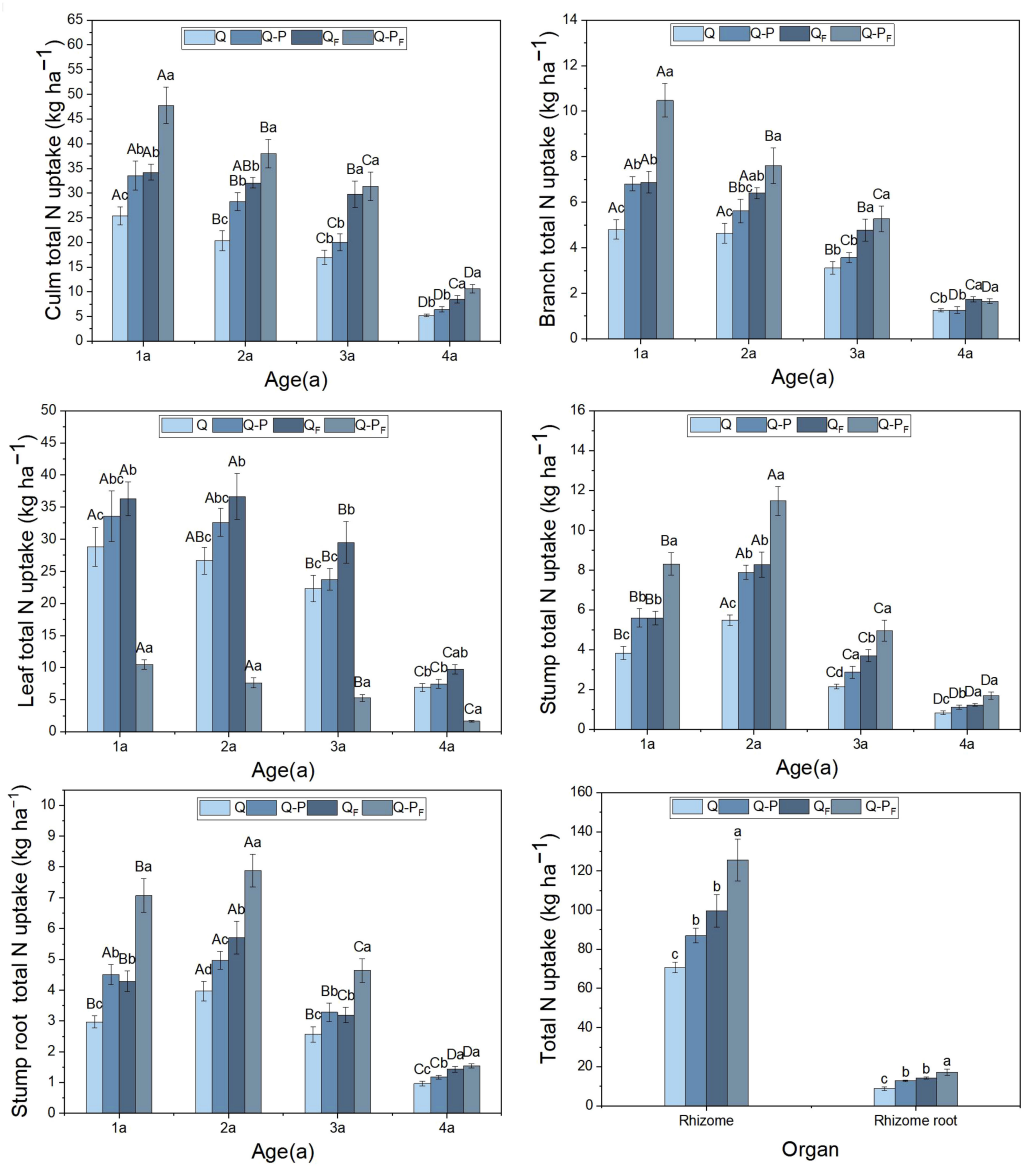
Organ nitrogen uptake of *Qiongzhuea tumidinoda* forests across four types of plots (Mean ± SD). In the first five figures, different uppercase letters in the same plot represent significant distinctions between different ages (*P< 0.05*), and different lowercase letters in the same age indicate significant differences between plots (*P< 0.05*). In the last figure, different lowercase letters within the same organ indicate significant differences between different plots (*P< 0.05*).

The differences in total nitrogen uptake among various organs of different ages in different types of bamboo were significant (*P< 0.05*, **Figure 4**), with all showing higher values for *Q. tumidinoda* mixed forest (Q-P) compared to pure *Q. tumidinoda* forest (Q). Compared to the unfertilized treatment, the nitrogen concentrations in various organs of both Q-P and Q types significantly increased under the fertilized treatment. Among them, the Q-P type showed a more pronounced increasing trend (average growth of 45.10%) than Q type (average growth of 42.41%). Nitrogen uptake for each organ (except rhizomes and rhizome roots) decreased with increasing bamboo age. Among these, leaves exhibited the highest total nitrogen uptake, accounting for an average of 29.33% (ranging from 28.31% to 31.52%) of the total uptake, and the aboveground parts showed a 31.52% higher total nitrogen uptake than that of the underground parts (*P< 0.05*). Q-P_F_ type displayed the highest total nitrogen uptake, reaching up to 478.41 kg ha^-1^.

### 3.3 Allocation of urea-^15^N in various forest types of *Q. tumidinoda* bamboo forests

Under fertilization, there was no significant difference in Ndff between Q-P and Q types (**Table 3**). There were significant differences in Ndff among different organs. The average Ndff of stump (0.25%) was the highest, while the average Ndff of branch (0.13%) was similar to that of culm (0.13%), with no significant difference (*P > 0.05*).

**Table 3:** Ndff of *Qiongzhuea tumidinoda* forests across various forest types (Mean ± SD).

Forest type	Age	Culm (100%)	Branch (100%)	Leaf (100%)	Stump (100%)	Stump root (100%)	Rhizome (100%)	Rhizome root (100%)
QF	1	0.17 ± 0.01Aa	0.17 ± 0.03Ab	0.26 ± 0.03Aa	0.31 ± 0.02Aa	0.32 ± 0.03Aa	0.15 ± 0.01a	0.16 ± 0.01a
	2	0.16 ± 0.02Aa	0.16 ± 0.02Ab	0.20 ± 0.01Bb	0.27 ± 0.03Ba	0.26 ± 0.02Bb		
	3	0.11 ± 0.01Ba	0.14 ± 0.02Aa	0.18 ± 0.01Ba	0.19 ± 0.02Ca	0.13 ± 0.02Ca		
	4	0.06 ± 0.01Ca	0.05 ± 0.01Ba	0.10 ± 0.01Cb	0.11 ± 0.01Da	0.06 ± 0.01Db		
Q-PF	1	0.19 ± 0.02Aa	0.22 ± 0.02Aa	0.27 ± 0.03Aa	0.32 ± 0.02Aa	0.46 ± 0.03Aa	0.20 ± 0.01a	0.21 ± 0.01a
	2	0.19 ± 0.02Aa	0.21 ± 0.01Aa	0.24 ± 0.03Ba	0.30 ± 0.03Aa	0.32 ± 0.01Aa		
	3	0.13 ± 0.01Ba	0.09 ± 0.01Ba	0.21 ± 0.02Ba	0.21 ± 0.03Ba	0.16 ± 0.01Ba		
	4	0.07 ± 0.01Ca	0.04 ± 0.01Ca	0.14 ± 0.02Ca	0.13 ± 0.01Ca	0.08 ± 0.01Ca		

Different lowercase letters for the same forest type indicate significant differences between different organs (*P*< 0.05). Different capital letters for the same forest type indicate significant differences between different ages (*P*< 0.05).

**Figure 5:**
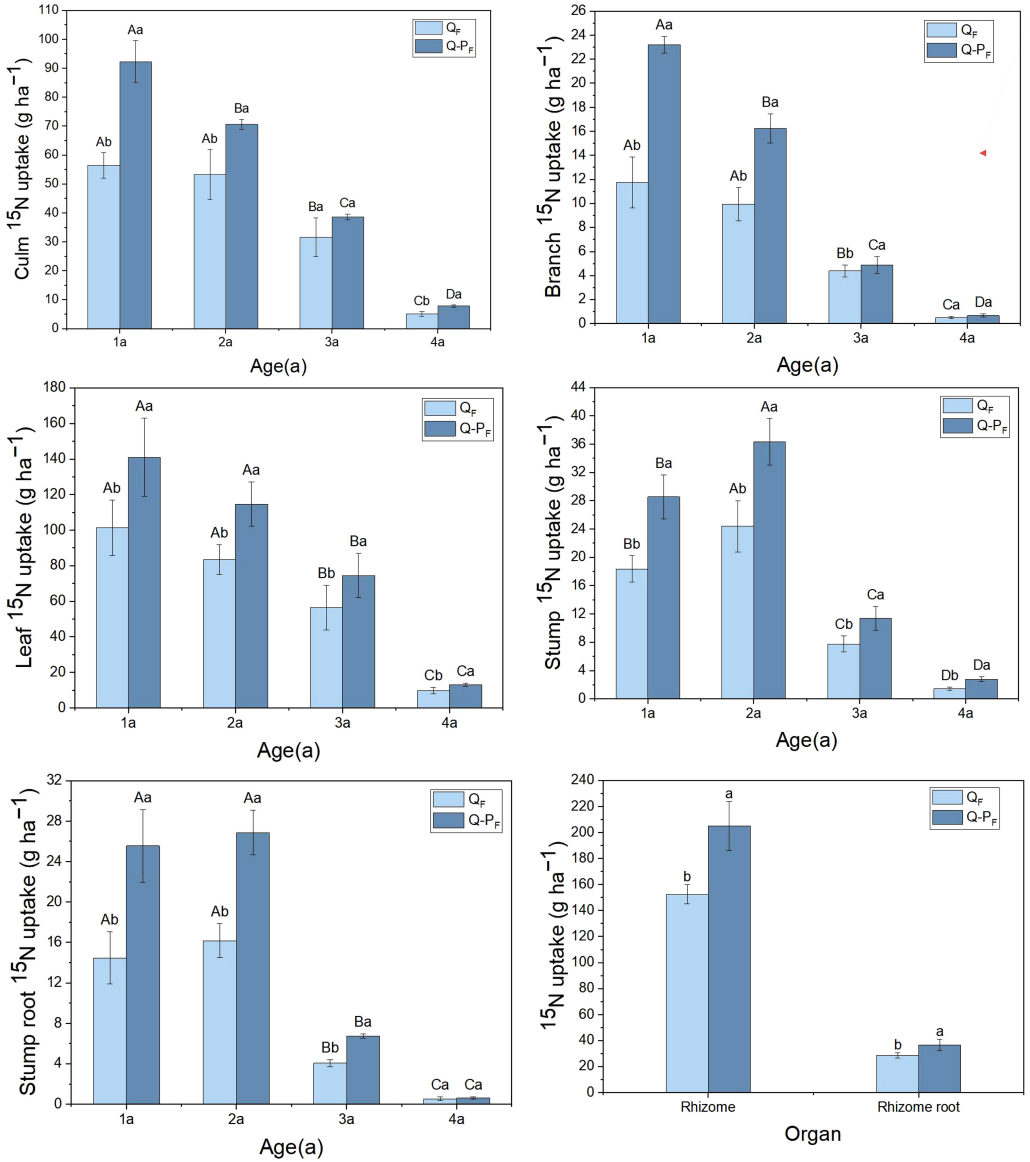
^15^N uptake of *Qiongzhuea tumidinoda* forests across four types of plots (Mean ± SD). In the first five figures, different uppercase letters in the same plot represent significant distinctions between different ages (*P< 0.05*), and different lowercase letters in the same age indicate significant differences between plots (*P< 0.05*). In the last figure, different lowercase letters within the same organ indicate significant differences between different plots (*P< 0.05*).

Significant differences were observed in the total ^15^N uptake among various organs of different types of *Q. tumidinoda* (**Figure 5**). The total ^15^N uptake in organs of Q-P type was notably higher than in Q type (*P< 0.05*). Except for culms and culm roots, most organs showed a decreasing trend in ^15^N uptake with increasing bamboo age. The total ^15^N uptake in leaves was notably higher than in other organs across both forest types, averaging 35.62% of the total uptake, ranging from 32.72% to 38.53%.

**Figure 6:**
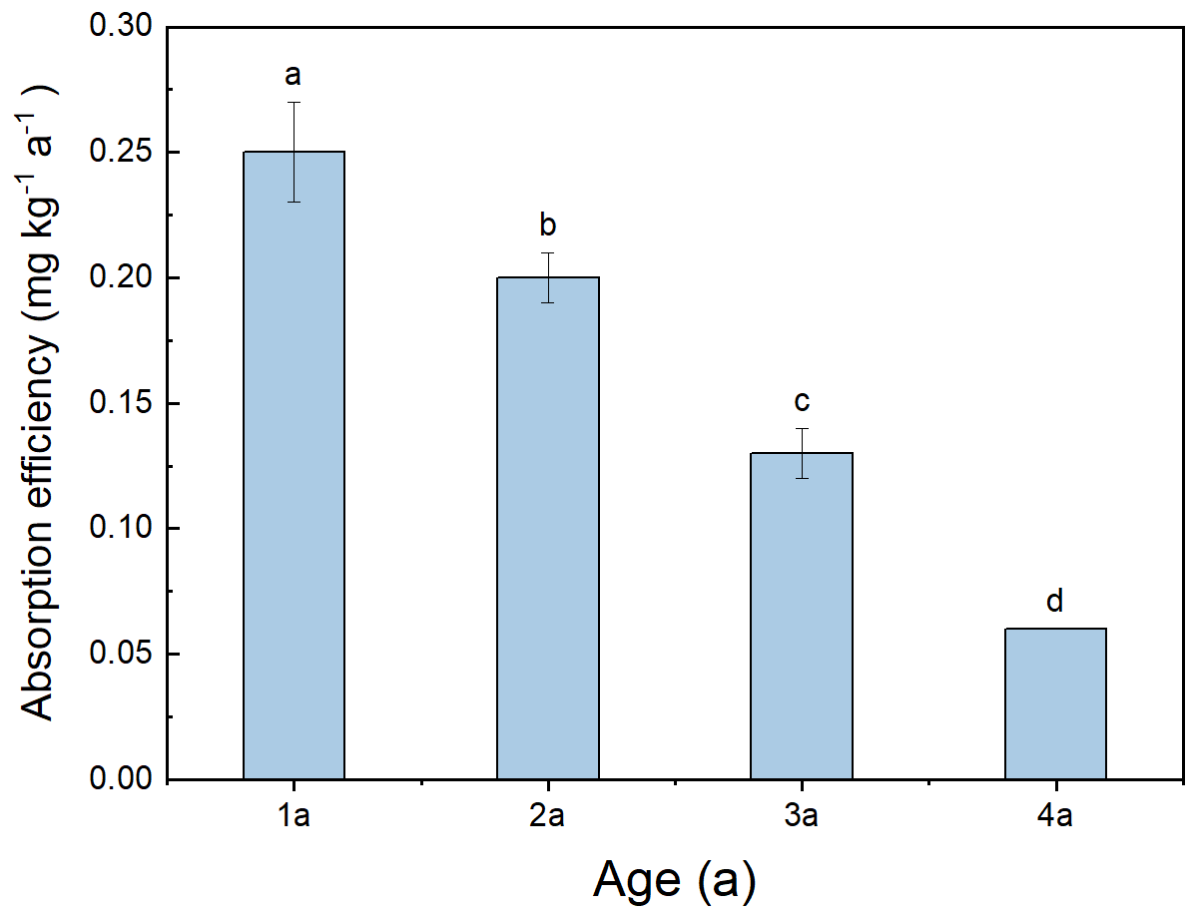
Total absorption efficiency of different ages in *Qiongzhuea tumidinoda* forests. Different lowercase letters represent significant differences between different ages (*P< 0.05*).

There were significant differences observed in the absorption of ^15^N among bamboo of different ages (**Figure 6**), with absorption efficiency gradually decreasing as bamboo aged. The absorption efficiency of 1a bamboo was notably higher than that of other ages, ranging from 0.20 to 0.28 (mean 0.25).

### 3.4 Allocation of residual urea-^15^N in soil

**Figure 7:**
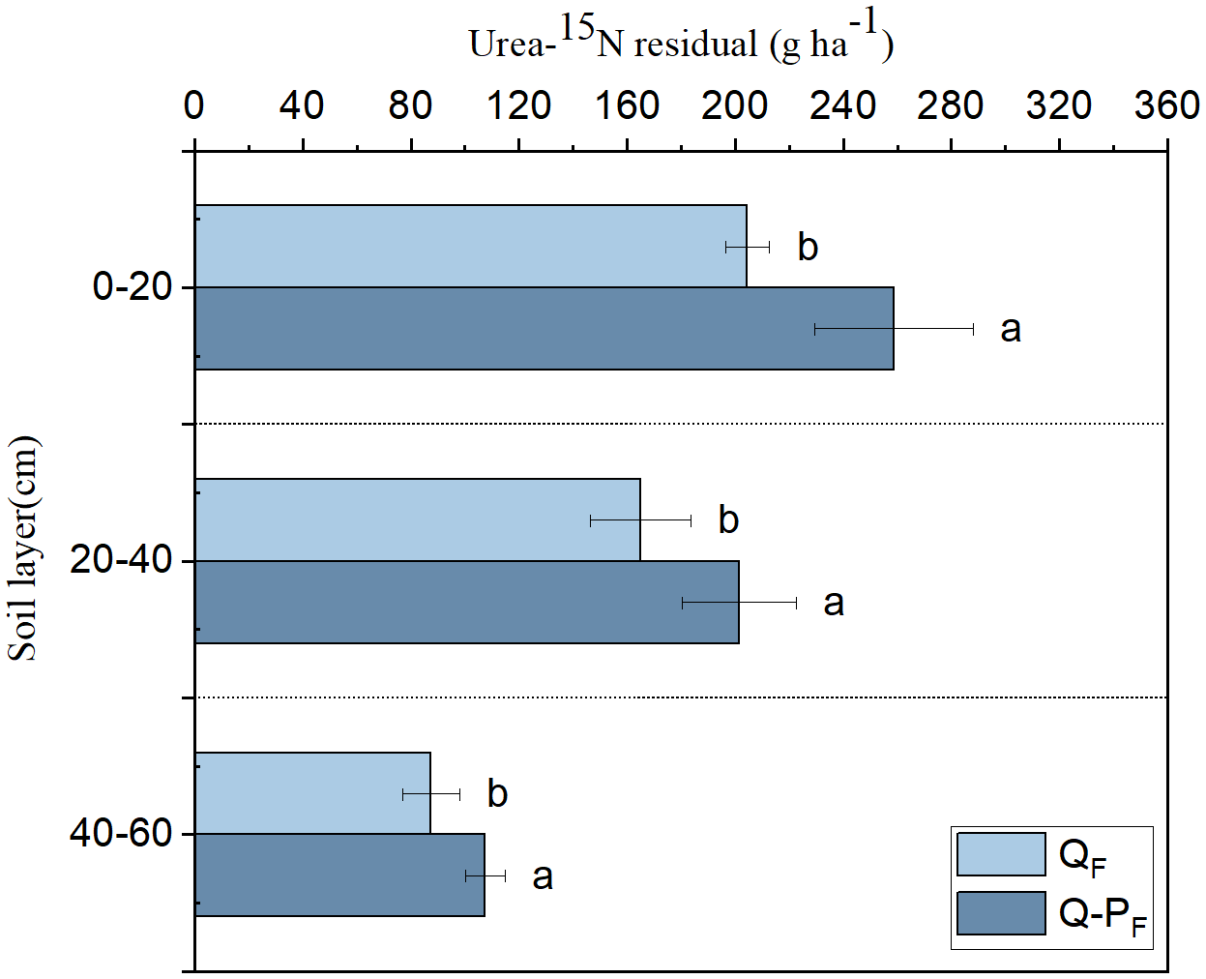
The distribution of residual urea-^15^N in the soil. Different lowercase letters of the same soil layer indicate significant differences among different sampling sites at the *P< 0.05* level.

In the same soil layer, Q-P type exhibited significantly higher total residual urea-^15^N compared to Q type (**Figure 7**). With increasing soil depth, there was a decreasing trend observed in the total residual urea-^15^N among different forest types. The majority of residual urea-^15^N was found in the 0–20 cm soil layer, accounting for 44.86% of the total residual in Q type and 45.56 in Q-P type, respectively.

### 3.5 Fate of urea-^15^N in bamboo-soil system

**Figure 8:**
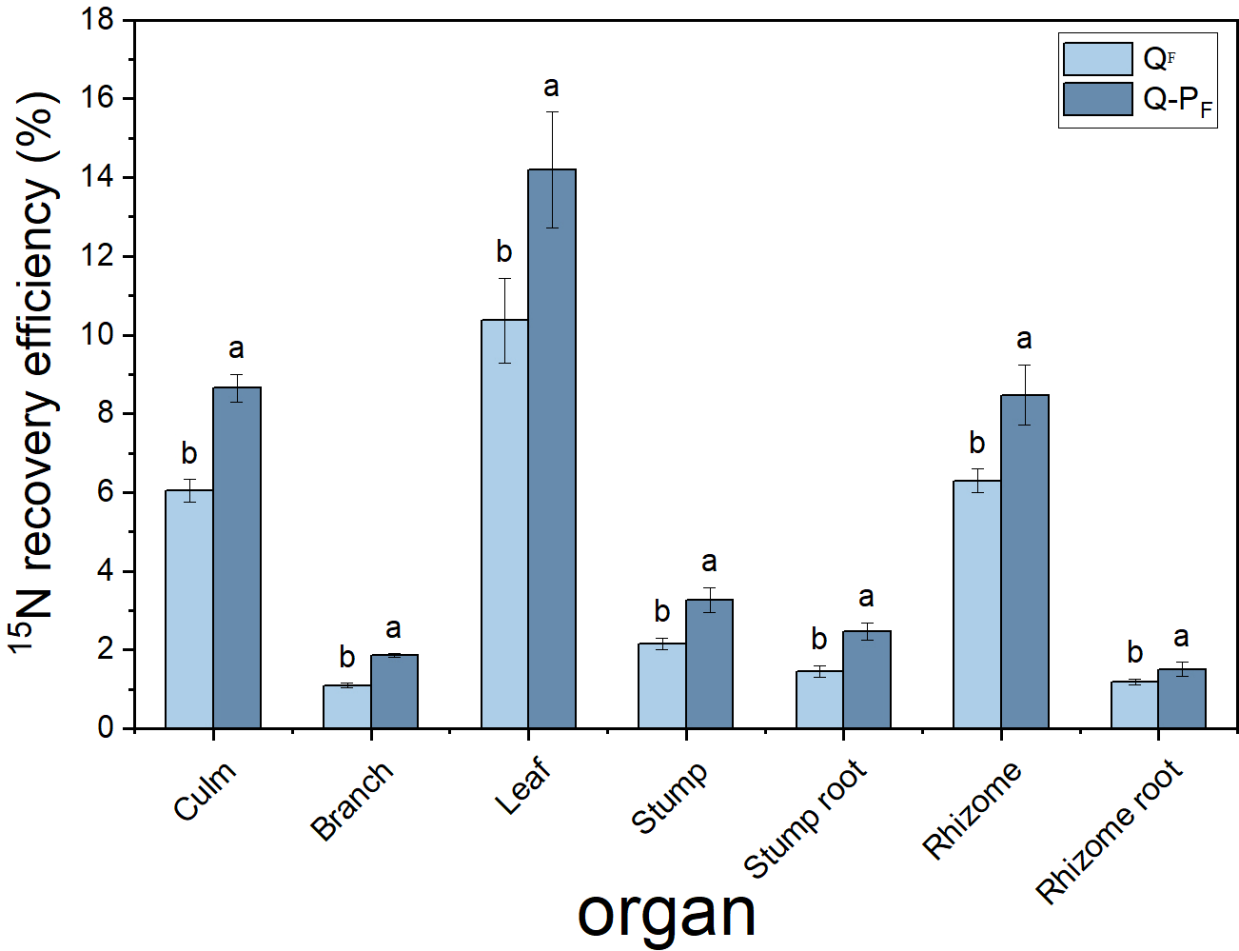
Effects of various forest types on ^15^N recovery efficiency in *Qiongzhuea tumidinoda* forests. Different lowercase letters within the same organ indicate significant differences between different sampling sites (*P< 0.05*).

**Figure 9:**
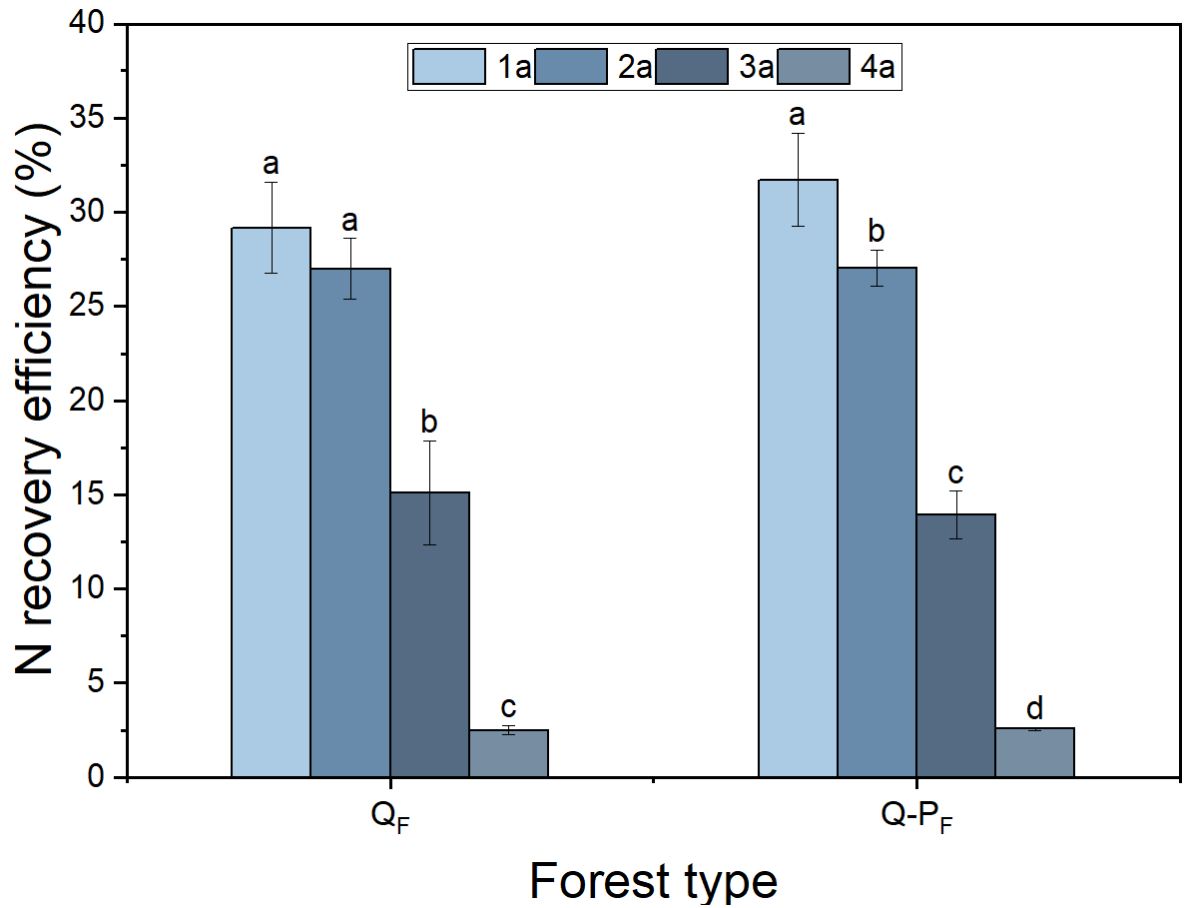
Effects of various forest types on the N recovery efficiency of different ages in *Qiongzhuea tumidinoda* forests. Different lowercase letters within the same forest type indicate significant differences in nitrogen recycling efficiency among different ages (*P< 0.05*).

**Figure 10:**
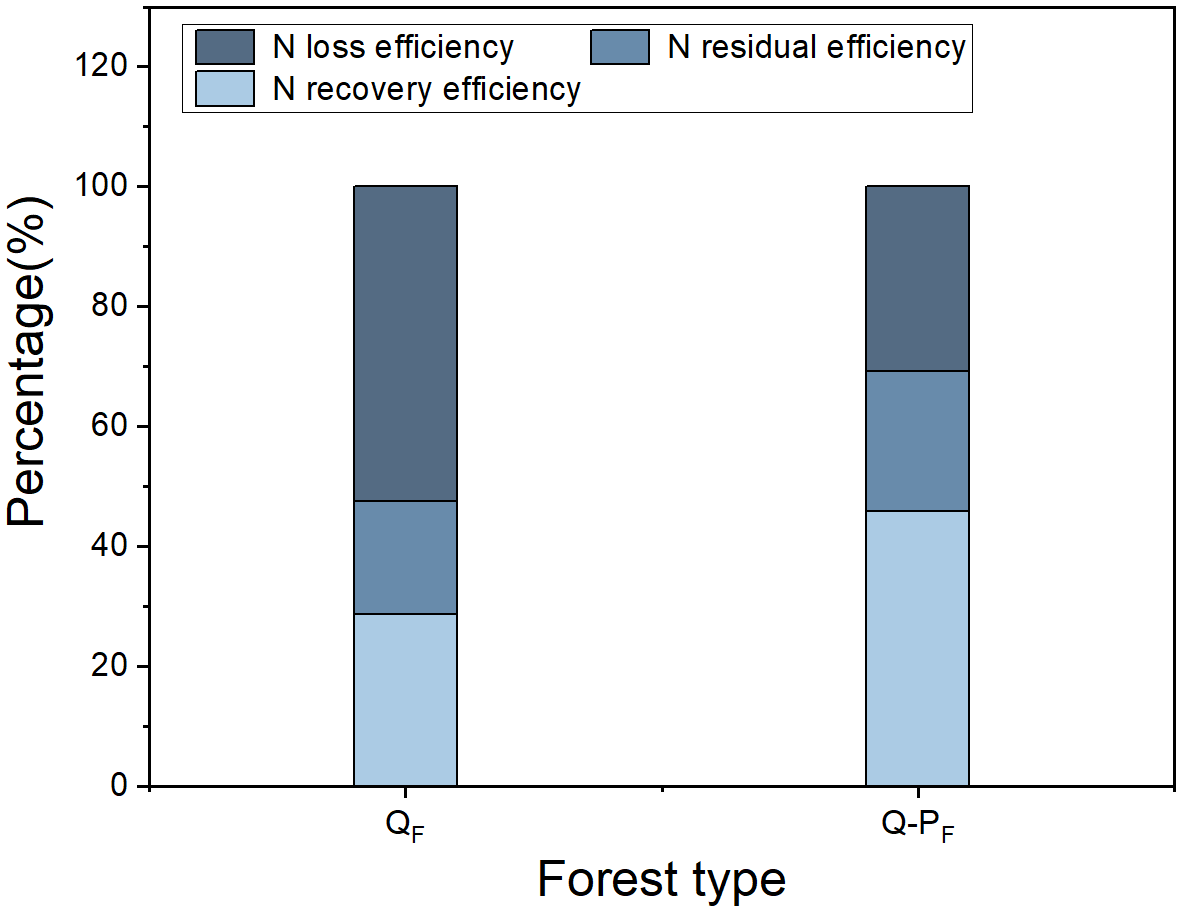
Effects of various forest types on the fate of urea-^15^N in *Qiongzhuea tumidinoda* forests.

There were significant differences observed in nitrogen recovery efficiency among different forest types, with Q-P type showing significantly higher nitrogen recovery efficiency in various organs compared to Q type. The ^15^N recovery efficiency of Q-P type culms, branches, leaves, stumps, and stump roots was significantly higher than that of Q type, with increases of 43.14%, 69.09%, 36.84%, 51.63%, 69.18%, 34.60%, and 26.89%, respectively. From an overall perspective, the nitrogen recovery efficiency of leaves averaged at 12.28%, ranging from 9.57% to 15.76%, notably higher than other organs. The culm roots exhibited the lowest recovery efficiency, ranging from 1.18% to 1.52% (**Figure 8**). The nitrogen recovery efficiency of Q-P and Q types at different ages showed no significant difference, but they exhibited the same pattern. That is, with the aging of bamboo, the nitrogen recovery efficiency significantly decreased (**Figure 9**). The nitrogen recovery efficiency and residual urea-^15^N were higher in Q-P type than in Q type, but nitrogen loss rate showed the opposite trend (**Figure 10**).

## 4 Discussion

Bamboo and broadleaved mixed forest is an excellent agricultural forestry model featuring bamboo. Research indicates that in competition with broadleaved trees, bamboo exhibits a greater advantage, possibly owing to its enhanced plasticity and environmental adaptability (Zheng and Lv, 2023). For instance, an experiment on nitrogen uptake conducted with *Castanopsis fargesii* and *moso* bamboo revealed that *moso* bamboo maintained dominance due to its higher tolerance threshold to ammonium nitrogen (Zou et al., 2020). Numerous studies indicated that a beneficial competition was established when bamboo was mixed with an appropriate proportion of broadleaved trees (Cao, 2001; Cheng et al., 2015; Yan et al., 2018). For example, research indicated that when the intercropping ratio of *moso* bamboo and broadleaved trees was in the range of 20–30%, optimal soil nutrients were achieved, leading to the best growth performance of *moso* bamboo (Zhang et al., 2020a). Similarly, The intercropping of *Q. tumidinoda* with other tree species had a certain impact on the growth of the bamboo forest, and the extent of this impact depended on the choice of tree species (Zhou et al., 2016).

Previous research indicated that *P. chinense* was one of the excellent native tree species for establishing *Q. tumidinoda* mixed forests. In the mixed forests of *P. chinense* and *Q. tumidinoda*, the diameter at breast height, height, and biomass of bamboo were significantly higher than those in pure *Q. tumidinoda* forests (Zhang et al., 2020b; Chen et al., 2022). This study also confirmed these findings, where the biomass of various parts of Q-P type’s *Q. tumidinoda* was significantly higher compared to Q type (except for rhizome and rhizome root). However, the difference in underground rhizome-root system (rhizome and rhizome root) between Q-P and Q types was not significant, possibly due to the occupation of certain underground spaces by *p. chinense* root. In the fertilized treatment, there was a significant difference in the underground rhizome-root system of Q-P type, indicating that compared to Q type, Q-P type’s rhizome-root system absorbed more nitrogen, consequently accumulating more biomass. Furthermore, compared to the unfertilized treatment, the biomass of various bamboo organs in both forest types increased under fertilization, but only the rhizome and rhizome roots reached significant levels, This could be attributed to the relatively high soil temperature and abundant rainfall during this period, which prompts bamboo to primarily focus its growth on the underground parts, accumulating a significant amount of nutrients in its rhizomes and shoots (Chen and Yang, 2003; Zhu et al., 2023).

**Figure 11:**
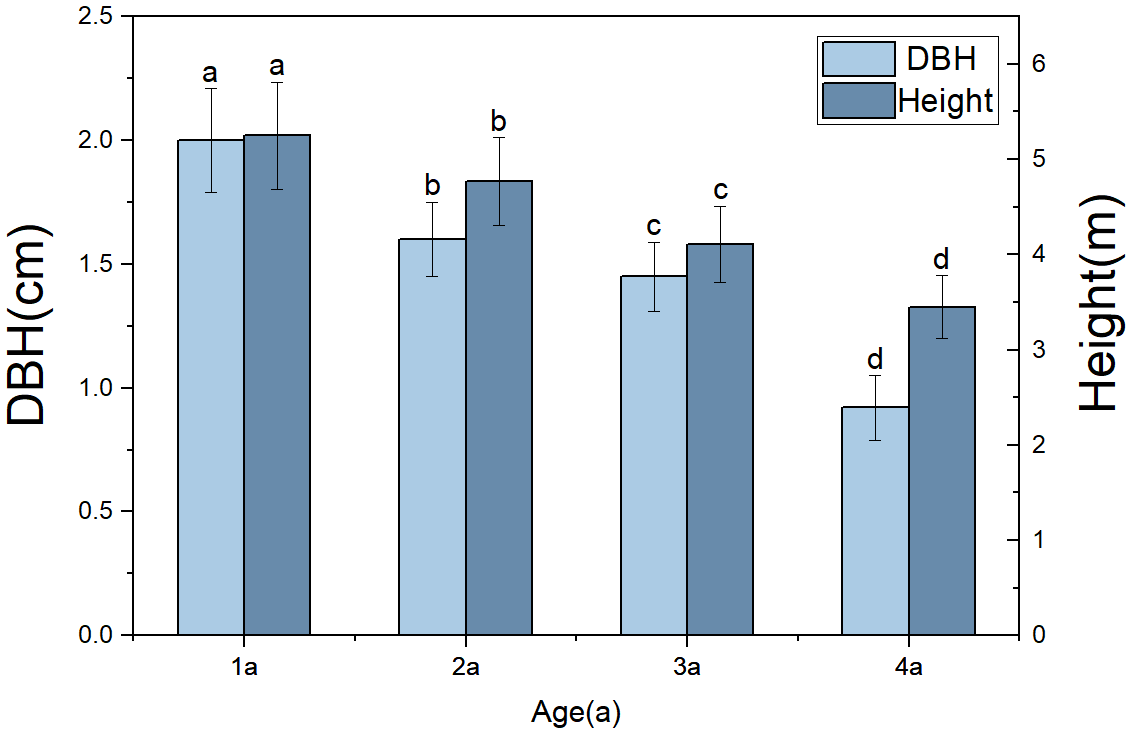
Comparison of Morphological Characteristics of *Qiongzhuea tumidinoda* in different forest types. The different lowercase letters of columns of the same color representing the height and diameter at breast height at different ages show significant differences (*P< 0.05*), respectively.

Due to age structure and individual size (**Figure 11**), the biomass of various organs of the four-year-old bamboo was significantly lower than in other age groups. The biomass of culm was the highest, consistent with previous research findings, accounting for 42.72% of the total biomass as revealed by this study (Chen et al., 2022).

Following fertilization, both the nitrogen concentration and uptake in various organs of Q-P type were significantly higher than in Q type, further emphasizing Q-P type’s greater nitrogen absorption. The leaves exhibited the highest nitrogen concentration and uptake, likely attributed to their photosynthetic activity. Despite the culms having the largest biomass, their nitrogen absorption was lower due to their comparatively lower nitrogen concentration.

In bamboo forest ecosystems, nitrogen fertilizer is primarily utilized in three ways: uptake by bamboo, retention within the soil, or loss from the bamboo-soil system (Chalk et al., 2015; Wang et al., 2023b). Currently, the use of ^15^N isotope tracing technology is considered the optimal method for studying nitrogen fertilizer utilization efficiency and nitrogen balance in bamboo forest ecosystems (Haque et al., 2022). This technique, employing labeled ^15^N fertilizers, enables direct or indirect determination of nitrogen recovery by bamboo, residual fertilizer levels in the soil, and nitrogen loss rates (Raymond et al., 2016; Su et al., 2019). In this study, ^15^N distribution varied among different types of *Q. tumidinoda* forests, yet the overall allocation pattern remained largely consistent. A range of 32.72% to 38.53% of the total ^15^N absorption was allocated to the leaves, representing the highest proportion. Following this, the branches accounted for 19.06% to 23.89% of the total ^15^N absorption. This alignment with the distribution of 26.90% to 37.21% of total ^15^N absorption in the leaves in *Moso* bamboo forests. However, discrepancies in the overall ^15^N absorption distribution were evident among different *Q. tumidinoda* forests. For instance, in *Moso* bamboo forests, bamboo stump ranked second (Su et al., 2019), indicating variations possibly attributed to different bamboo species. Based on previous studies, the residual amount and downward movement of nitrogen fertilizer can be reflected by the concentration of ^15^N in the soil layers (Wang et al., 2011; Jing et al., 2020). The average residual amount of ^15^N-labeled urea in the 0–60 cm soil layer of Q-P type was 567.73 g ha^-1^, accounting for 23.46% of the total applied ^15^N. It was significantly higher in all soil layers compared to Q type, correlating with the soil organic matter content. Research has shown that nitrogen becomes immobilized within soil organic matter (Mostafa et al., 2020). In this study, the participation of *p. chinense* ‘s litter in decomposition led to higher organic matter content in all soil layers of Q-P type compared to Q type, consequently immobilizing more nitrogen. The residual ^15^N in both forest types exhibited a decreasing trend with increasing soil depth, consistent with previous reports (Ru et al., 2020; Nguyen et al., 2021; Effah et al., 2022).

**Figure 12:**
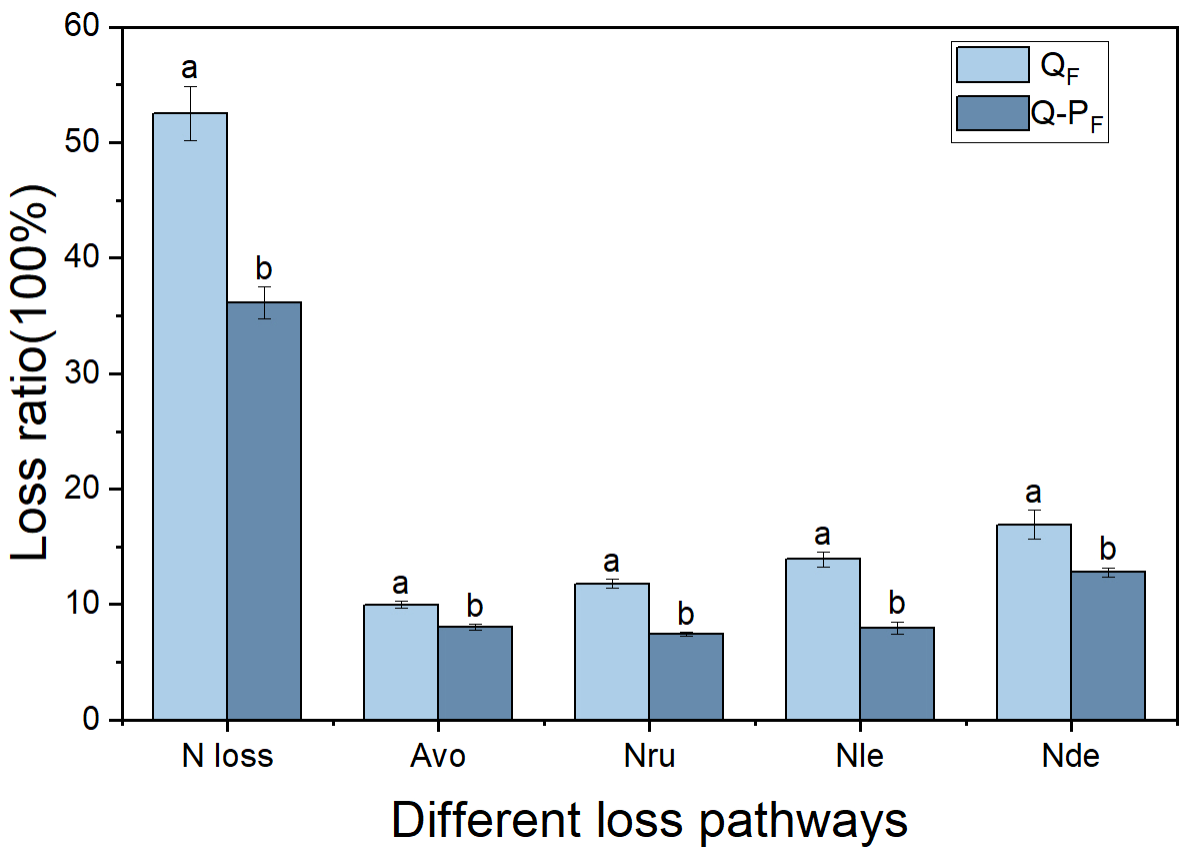
Comparison of nitrogen loss rates through various pathways of *Qiongzhuea tumidinoda* in different forest types. Different lowercase letters within the same forest type indicate significant differences in nitrogen loss rates through different nitrogen loss pathways (*P< 0.05*), respectively. Avo, ammonia volatilization; Nru, nitrogen runoff; Nle, nitrogen leaching; Nde, nitrification-denitrification.

It is well-known that ammonia volatilization, nitrification-denitrification, runoff, and leaching are the primary pathways for nitrogen loss (Räbiger et al., 2020; Shi et al., 2020; Lan et al., 2022). In this study, it was found that the nitrogen recovery efficiency and soil nitrogen residual efficiency of the mixed forest (Q-P) were significantly higher than those of the pure forest (Q), which may be attributed to the differences in nitrogen loss between them. All types of nitrogen loss pathways in the Q-P type (including ammonia volatilization, runoff, leaching, and nitrification-denitrification) were significantly higher than those in the Q type (**Figure 12**). This conclusion can be inferred from previous studies. For instance, research has indicated that compared to pure bamboo forests, the canopy of mixed bamboo and broadleaf tree forests provides effective shading, thus reducing ammonia volatilization (Cheng et al., 2015; Zhang et al., 2020a). Additionally, the canopy interception by broadleaf trees in mixed forests, along with the impact of their litter and root systems on soil and water conservation (Zhang et al., 2020a; Nainar et al., 2021; Ding et al., 2023), results in lower nitrogen losses from runoff and leaching compared to pure forests. Apart from differences in nitrogen loss, the Q-P type exhibited two distinct nitrogen recovery pathways: the recovery rate of ^15^N from *P. chinense* (5.37%, **Table 4**) and the recovery rate of ^15^N from bamboo (40.43%). Compared to the single forest type of Q type, Q-P type more efficiently utilized the abundant nitrogen resources.

**Table 4:** Biomass allocation characteristics and nitrogen fate of *phellodendron chinense* forests in Q-P (Mean ± SD).

Organ	Biomass (t ha^-1^)	Nitrogen concentration (g kg^-1^)	Nitrogen uptake (kg ha^-1^)	^15^N uptake (g ha^-1^)	Nitrogen recovery efficiency (%)
Trunk	8.87 ± 1.26	7.23 ± 0.38	64.00 ± 8.10	57.60 ± 7.29	2.38 ± 0.30
branch	4.29 ± 0.77	10.23 ± 0.94	43.45 ± 3.95	39.10 ± 3.56	1.61 ± 0.15
Leaf	0.05 ± 0.01	19.09 ± 1.38	0.91 ± 0.19	0.82 ± 0.17	0.03 ± 0.01
Root	1.67 ± 0.20	21.90 ± 1.70	36.38 ± 24.11	32.72 ± 2.86	1.35 ± 0.12

In this study, the mean nitrogen recovery rate for both bamboo forest systems was 37.21%, significantly higher than the nitrogen recovery rate determined by Su et al (Su et al., 2019). in fertilized Moso bamboo forests (28.98%). These findings differed from the recovery rate of fertilization in Moso bamboo forests reported by Mao et al (Mao et al., 2016). (13.96%). This discrepancy could potentially stem from variations in bamboo biological characteristics, timing, and dosage of fertilizer applications. Therefore, further experiments are needed to validate the specific reasons.

## 5 Conclusions

In this study, it was clearly indicated that there are significant differences in the fate and distribution ratios of nitrogen labeled urea applied in different types of bamboo forest ecosystems. The bamboo forest with mixed *P. chinense* and *Q. tumidinoda* exhibited notably higher nitrogen recovery and soil residue compared to the pure *Q. tumidinoda* forest, while showing an opposite trend in nitrogen loss rate. In these two types of bamboo forests, despite the largest biomass being in the bamboo culms, the leaves exhibited the highest nitrogen absorption and content. The residual ^15^N was primarily concentrated in the fertilized layer. These studies indicate that the proportion of trees to bamboo in this experimental design may fall within an appropriate range of mixed cropping ratios, thereby enhancing the nitrogen recovery efficiency of bamboo and reducing nitrogen loss efficiency. However, the competitive relationship between trees and bamboo cannot be ignored. Therefore, we hypothesize that increasing or decreasing the proportion of trees in bamboo forests may have similar or opposite effects on the nitrogen cycle of bamboo, which requires further experimental support. Additionally, the impact of different types of mixed tree species on bamboo may vary, especially the intercropping of nitrogen-fixing tree species with bamboo, which will be the focus of future research.

## Data availability statement

The raw data supporting the conclusions of this article will be made available by the authors, without undue reservation.

## Author contributions

YW: Formal analysis, Investigation, Writing – original draft, Writing – review & editing, Conceptualization, Methodology. WD: Conceptualization, Funding acquisition, Supervision, Writing – review & editing. HZ: Methodology, Writing – review & editing. JD: Data curation, Investigation, Writing – review & editing. WL: Data curation, Investigation, Writing – original draft. CP: Formal analysis, Writing – original draft. XL: Formal analysis, Writing – original draft. ZX: Project administration, Resources, Writing – review & editing.
